# On the Local Treatment of Erysipelas

**Published:** 1883-12-20

**Authors:** John Kent Spender

**Affiliations:** M.D., London; Physician to the Mineral Water Hospital, Bath


### ON THE LOCAL TREATMENT OF ERYSIPELAS.

By John Kent Spender, M.D., Lond.,
Physician to the Mineral Water Hospital, Bath,

This subject deserves every attention, and the readers of the British Medical Journal ought to be thankful that it has been so often discussed. But the question of etiology is, perhaps, not always clearly grasped, although it must determine to a large extent the type of the disease, and its successful management. Take erysipelas of the scalp, for instance; a superficial erysipelas is a very innocent thing, and easily controlled; but there is hardly a more dangerous malady than traumatic erysipelas of this part, accompanying profuse suppuration in the connective tissue between the occipito-frontalis muscle and the cranium. Again, if the sanitary arrangements of a private house or of a large public institution be defective, there may be frequent epidemics of facial erysipelas and of enteric fever, either separately or conjointly, as happened in the Somerset Lunatic Asylum in 1879 and 1881.

But if surgical and hygienic agencies can be safely put aside, it may be acknowledged that the local treatment of erysipelas deserves a large amount of professional care and discrimination. Call it by what name we like, it is essentially a spreading dermatitis, which may cause peril by the extent of cutaneous surface involved, or by the degree of constitutional irritation which may be provoked. It is not often that we have a quasi-inflamation so completely under therapeutic command. Iodine may be useful if there be any suspicion of a pyæmic complication; but, for the so-called idopathic erysipelas, I cannot speak too highly of the free and frequent application of a solution of tannin in equal parts of spirits of wine and water, as recommended by Dr. Braithwaite in the Journal for April 30th, 1881 This solution is quite as beneficial when erythema approaches erysipelas in local and general severity. I give the bare outlines of two cases:

1. A lady, a little past middle age, had a sudden attack of erysipelas all over the left thigh and leg after a trivial injury. The general health was tolerably good. About a dozen "paintings" with the solu ion of tannin were sufficient to drive away every trace of the disease. The skin swollen presented a shrivelled look.

2. A maiden lady in middle life, entrusted to my care by Mr. Clouting, of Thetford, suffered from erratic erysipelas on the face, after exposure to cold, in October, 1881. The tannin solution was very successful, and a recent letter from Mr. Clouting tells us that the lotion has been frequently used during the last twelvemonth with the same good resalt.

Tannin completely dissolves in equal parts of water and spirits of wine; and, when applied to the skin with a camel's hair brush, a delightfully cool feeling follows from evaporation. A proper strength is six grains to the drachm of fluid.

One of the great literary wants of our profession is, a first-rate monograph on erysipelas in its medical and surgical aspects. Just because it has these two aspects, the subject has rather "fallen between two stools," although handled with more or less ability in various dictionaries and encyclopædias. But there are several points in its pathology and treatment on which most medical men would like to have new and trustworthy teaching.—*British Med. Journ., Dec. 9, 1882, p. 1147.*

